# Le médulloblastome chez l'adulte: à propos de 13 cas et revue de la literature

**DOI:** 10.11604/pamj.2015.22.126.7242

**Published:** 2015-10-12

**Authors:** Jamal Drissi, Mariam Affane, Abdelhamid Elomrani, Mouna Khouchani

**Affiliations:** 1 Service d'Oncologie-Radiothérapie, CHU Mohamed VI, Marrakech, Maroc

**Keywords:** Médulloblastome, adulte, chirurgie, radiothérapie, Medulloblastoma, adult, surgery, radiotherapy

## Abstract

Le médulloblastome est une tumeur neuro-ectodermique primitive maligne. Il s'agit d'une tumeur rare chez l'adulte, représentant moins de 1% des tumeurs cérébrales. Nous proposons une étude rétrospective réalisée au sein du service d'Oncologie-Radiothérapie du CHU Mohamed VI de Marrakech sur une période de 13 ans. Le but de notre travail est de déterminer le profil épidémioclinique, thérapeutique, évolutif ainsi que les facteurs pronostiques de cette entité pathologique avec une revue de la littérature. Notre série comportait 13 patients, 10 hommes et 3 femmes, l’âge médian a été 20,8 ans. Le tableau clinique a été révélé par un syndrome d'hypertension intracrânienne (100%), associée à un syndrome cérébelleux (84%). La localisation était hémisphérique (31%) et vermio-hémisphérique (54%). 31% des patients ont bénéficié d'une exérèse chirurgicale totale. 85% des cas avaient une variante classique et 15% une variante desmoplasique. 30% des cas avaient été classés à «risque standard» et 70% à «haut risque». La chirurgie avait été complétée d'une radiothérapie de l'ensemble du névraxe selon la technique de «jonctions mobiles» dans tous les cas. Le délai moyen était de 73 jours. Une chimiothérapie adjuvante avait été réalisée chez 9 cas. Avec un recul moyen de 21.3 mois, l’évolution a été marquée par une récidive tumorale (4 cas), une toxicité auditive (6 cas) et des troubles cognitifs chez un cas. La prise en charge du médulloblastome doit être multidisciplinaire associant neurochirurgiens et oncologues radiothérapeutes. Cette collaboration est le seul garant d'une amélioration de son pronostic.

## Introduction

Le médulloblastome est une tumeur neuro-ectodermique primitive maligne (primitive neuro-ectodermic tumor PNET) [1], grade IV histologique selon la classification de l'OMS [2], très fréquente chez l′enfant. Par contre, il s′agit d′une entité rare chez l'adulte, représentant moins de 1% des tumeurs cérébrales [3, 4]. En conséquence, le médulloblastome chez l'adulte reste une pathologie mal connue. Notre étude a pour but de déterminer le profil épidémioclinique, thérapeutique et évolutif ainsi que les facteurs pronostiques de cette entité pathologique à Marrakech avec une revue de la littérature.

## Matérials et Méthodes

Il s'agit d'une étude rétrospective de 13 cas de médulloblastome chez l'adulte (âge ≥ 16 ans) prise en charge au service d'Oncologie-Radiothérapie du Centre Hospitalier Universitaire Mohamed VI de Marrakech entre janvier 2002 et décembre 2014. On a inclus les dossiers qui avaient une confirmation histologique. Le recueil des données a concerné tous les éléments cliniques, paracliniques, thérapeutiques et évolutifs.

## Résultats

**Figure 1:**
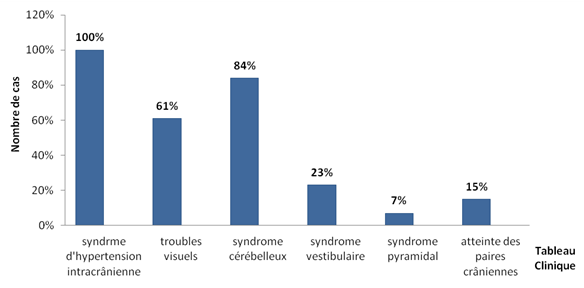
Répartition des patients selon le tableau Clinique

**Figure 2:**
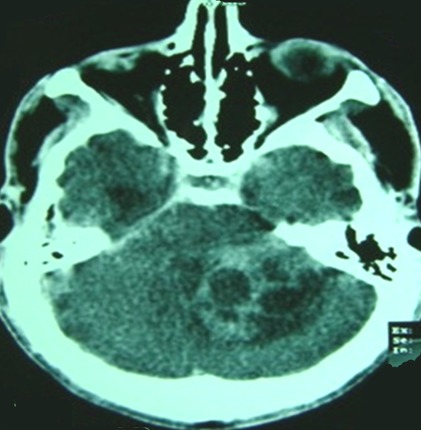
Scanner cérébral en coupe axiale avec injection du produit de contraste qui montre un processus tumoral hémisphérique gauche prenant le contraste de façon homogène associé à des hypodensités intratumorales

**Figure 3:**
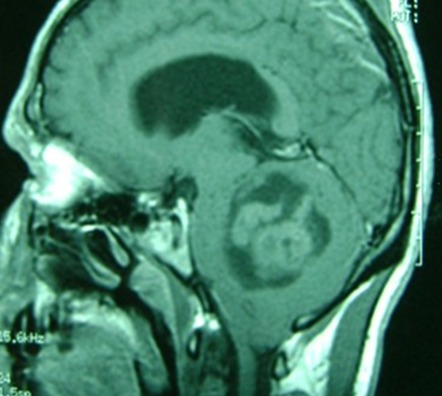
IRM cérébrale en coupe sagittale séquence pondérée T1 avec gadolinium qui montre un processus tumoral cérébelleux avec rehaussement modéré associe à un œdème périlésionnel et responsable d'une compression du tronc cérébral en avant

**Figure 4:**
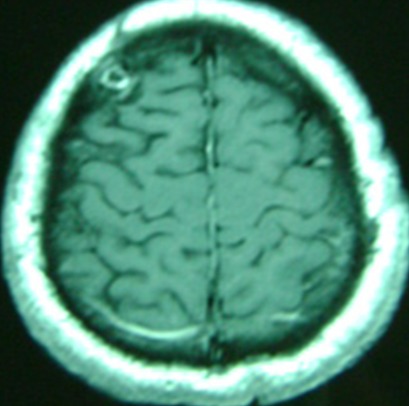
IRM cérébrale en coupe axiale séquence T1 avec gadolinium qui montre une lésion supracentimétrique frontale droite prenant le contraste en périphérie compatible avec une métastase

L’âge médian des cas a été de 20,8 ans (extrêmes de 16 à 33 ans), avec une prédominance masculine (sexe ratio de 3.33). La durée d’évolution des symptômes était entre 1 et 12 mois (moyenne de 4,6 mois). La tumeur était révélée par un syndrome d'hypertension intracrânienne (100%) fait de céphalées (100%), vomissements (76%) et de troubles visuels (61%), suivi par un syndrome cérébelleux (84%) de type stato-kinétique chez 10 cas et statique chez 1 cas, puis un syndrome vestibulaire (23%), un syndrome pyramidal (7%), atteinte des paires crâniennes (15%) (Figure 1). L'indice de Karnofsky moyen était de 70%. Nous avons noté une association avec une maladie de Von Recklinghausen chez un cas et une grossesse de 16 semaines d'aménorrhées révolues chez une patiente. Une tomodensitométrie a été réalisée chez 9 cas, elle a montré une tumeur spontanément hyperdense dans 10 cas, avec rehaussement après injection de produit de contraste dans tous les cas, une hypodensité intratumorale dans 3 cas et un œdème périlésionnel dans 3 cas (Figure 2). Une imagerie par résonnance magnétique a été réalisée chez 7 cas, elle a montrée une tumeur spontanément hypointense en séquence T1 et hyperintense en séquence T2, se rehaussant de façon assez homogène par l'injection de produit de contraste paramagnétique dans tous les cas (Figure 3). Sur les deux examens, la tumeur était hémisphérique (31%), vermio-hémisphérique (54%) et vermienne (15%), la taille moyenne était de 57 mm, la tumeur était associée à une hydrocéphalie dans 9 cas, une infiltration du 4^ème^ ventricule (5 cas), du tronc cérébral (3 cas) ainsi qu'une métastase frontale (1 cas) (Figure 4). Le traitement avait comporté trois volets: médical, chirurgical et adjuvant. Le traitement médical a été à base d'antalgique et de corticothérapie pour lutter contre l'hypertension intracrânienne. Le traitement chirurgical a comporté la réalisation d'une dérivation du liquide céphalorachidien (9 cas) puis l'exérèse chirurgicale de la tumeur par voie sous-occipitale médiane chez 8 cas et par voie sous occipitale latérale chez 5 cas. L'exérèse était macroscopiquement totale dans 4 cas, subtotale dans 3 cas, partielle dans 5 cas et une simple biopsie chez 1 cas. L’étude histologique avait montré une variante classique dans 85% des cas et desmoplasique dans 15% des cas. Au décours d'un bilan d'extension fait d'une imagerie craniospinale postopératoire et d'un examen du liquide céphalorachidien, 30% des cas étaient classés de «risque standard» et 70% de «haut risque». La chirurgie était complétée d'une radiothérapie de l'ensemble du névraxe comportant une irradiation à dose de 54 Gy en 30 fractions de 1,8 Gy sur la fosse postérieure et de 23.4 - 36 Gy en 13-20 fractions de 1,8 Gy craniospinale dans tous les cas, avec un complément focal par radiothérapie sur une métastase frontale dans un cas. Cette irradiation était réalisée selon la technique de «jonction mobile». Le délai moyen entre la chirurgie et la radiothérapie était de 73 jours. Une chimiothérapie adjuvante était réalisée chez 9 cas selon le protocole PACKER 99. Avec un recul moyen de 21.3 mois, l’évolution à long terme était marquée par une récidive tumorale chez 4 cas après un délai moyen de 19,5 mois (entre 9 et 38 mois). Concernant la toxicité, nous avons noté une toxicité auditive chez 6 cas, des troubles cognitifs chez un cas.

## Discussion

Le médulloblastome appartient à la famille des tumeurs primitives neuroectodermiques (PNET) [1]. Il reste exceptionnel chez l'adulte, représentant moins de 1% des tumeurs cérébrales [3, 4], avec une incidence annuelle <0,05/100 000 habitants [5]. L’âge médian de 20,8 ans est proche de celui des études de la littérature (entre 26 ans et 31 ans) [3, 5, 6] et la différence peut-être expliqué par la taille réduite de notre série et la différence dans la définition de population «adulte» entre les équipes. Une prédominance masculine était retrouvée dans notre série comme dans la majorité de celles de la littérature [3, 5, 6]. Bien que la plupart des cas sont sporadiques, quelques facteurs de risque étaient rapportés dans la littérature; ils peuvent être congénitaux (syndrome de Turcot, syndrome de Rubinstein-Taybi, syndrome de Von Recklinghausen et syndrome de Li-Fraumeni) [3] ou environnementaux (radiations ionisantes et certains virus), un cas de notre série a présenté un syndrome de Von Recklinghausen.

Cliniquement, le médulloblastome se traduit par un syndrome d'hypertension intracrânienne qui est présent dans 60% à 93% des cas [6–8]. Dans notre série, en comparaison avec les autres séries du tiers-monde [4, 6, 8], ce syndrome était présent dans 100% des cas et pourrait s'expliquer par la durée d’évolution longue (durée moyenne de 4,6 mois). Les signes en rapport direct avec l'atteinte cérébelleuse vermienne se traduisent par des troubles de l’équilibre et de la marche (syndrome cérébelleux statique). Chez l'adulte, les signes cliniques peuvent être en rapport avec une atteinte latéro-cérébelleuse et se traduire avant tout par des troubles de la coordination (syndrome cérébelleux cinétique) [3, 6, 8]. Un syndrome vestibulaire est présent dans 23% des cas [6]. Dans 20% des cas est notée une atteinte des paires crâniennes (par ordre de fréquence VI, V, VII, VIII, III). Le médulloblastome est la tumeur intracérébrale qui a le plus de propension à donner des métastases avant tout leptoméningées et parfois intraparenchymateuses. Elles sont surtout observées au niveau de l'encéphale sous la forme de lésions superficielles nodulaires et peuvent se traduire cliniquement par des troubles généraux cognitifs ou focaux [9]. Les localisations spinales peuvent se manifester par des signes médullaires et/ou radiculaires.

A l'aspect scanographique, le médulloblastome est décrit comme une masse hyperdense (66%) [10], bien limitée, avec rehaussement après injection du produit de contraste, des aspects atypiques (hypodensité intratumorale, calcifications, œdème périlésionnel) sont possibles. Les auteurs s'accordent sur la prédominance de la localisation latérale chez l'adulte (approximativement 50%) [6, 7, 11]. L'imagerie par résonance magnétique est l'examen clé à demander pour l'exploration de la fosse cérébrale postérieure, elle montre une masse bien limitée en hyposignal T1 et en hypersignal T2 avec un rehaussement modéré et hétérogène après injection du gadolinium. Les formes histologiques les plus souvent rapportées sont la variante classique et desmoplasique. En comparaison avec l'enfant, cette dernière est fréquente chez l'adulte (30% à 40%) [4, 6, 11]. Dans notre série, 20% des patients avaient un médulloblastome desmoplasique.

Le médulloblastome sera classé par la suite sur la base des données clinico-chirurgicales en deux groupes ayant une implication thérapeutique: groupe à «risque standard» et groupe à «haut risque» [5].

L'exérèse chirurgicale est indispensable au diagnostic anatomopathologique et comme première phase du traitement. Elle doit être radicale chaque fois que cela est possible. Dans notre série, 30% d'exérèses étaient macroscopiquement totales, ce qui semble comparable à certaines études [8, 11]. Avec l'amélioration des techniques opératoires et de réanimation, les complications et la mortalité opératoire sont devenues négligeables par rapport à autrefois. Le médulloblastome est une tumeur hautement radiosensible. Sa grande propension à disséminer dans l'ensemble du système nerveux central aboutissant aux échecs de la radiothérapie focalisée sur la fosse postérieure, ont conduit à réaliser une radiothérapie de l'ensemble du névraxe [9]. Le traitement est délivré en procubitus aligné, horizontalisé avec une immobilisation par des systèmes de contention personnalisés. Elle comporte donc l'irradiation du site primitif au niveau de la fosse cérébrale postérieure ainsi qu'une irradiation prophylactique de l'ensemble du système nerveux central associant classiquement 54 Gy en 30 fractions de 1,8 Gy sur la fosse postérieure et 23.4 à 36 Gy en 12 à 18 fractions de 1,8 Gy sur le névraxe. Les métastases cérébrales et spinales peuvent bénéficier d'une irradiation focale. Cette irradiation est réalisée le plus souvent selon la technique «de jonctions mobiles» qui permet de limiter les risques de surdosage de l'axe médullaire et de sous-dosage des espaces sous-arachnoidiens [9]. Les nouvelles techniques de radiothérapie, telles que la tomothérapie et la protonthérapie, sembleraient optimiser la dosimétrie et apporter un gain dans la protection des organes à risque. Ce traitement doit être commencé le plus tôt possible et le délai de sa réalisation ne doit pas être retardé au-delà de 90 jours. Dans notre série, le délai moyen entre la chirurgie et le début de la radiothérapie était de 73 jours certainement à cause du contexte socio-économique. Chez l'enfant, un tel traitement permet d'obtenir une survie sans maladie à long terme de 65% environ [12]. Les complications de la radiothérapie ont conduit à améliorer la technique en diminuant la dose et en modifiant le volume cible. L'association d'une irradiation crâniospinale à dose réduite à une chimiothérapie permet d'améliorer le pronostic avec une survie sans maladie à cinq ans de 75-80%. Le rôle de la chimiothérapie chez l'adulte est toujours discutable [5, 6, 11, 13, 14], elle peut être indiquée dans les groupes à «haut risque» et en cas de récidive tumorale, et doit être évaluée dans les groupes à «risque standard».

L’évolution peut se faire par deux modes; La récidive locale au niveau de la fosse cérébrale postérieure constitue le mode de rechute le plus fréquent, cette rechute peut apparaître tardivement (50% après 2 ans, 20% après 5 ans) [6, 14]. Riffaud a rapporté un cas de récidive tumorale après 18 ans [7]. La métastase intranévraxique ou somatique (os, ganglions lymphatiques, poumons,…), des cas rares de dissémination intra-péritonéale via une dérivation ventriculo-péritonéale ont été rapportés, elles sont évitées par l'incorporation de filtres millipores. La survie globale est actuellement meilleure qu'autrefois; la survie à 5 ans peut atteindre 80% [5, 7, 14] et varier entre 40% et 60% après 10 ans [7, 13]. Parallèlement, ces traitements sont extrêmement pourvoyeurs de séquelles à long terme. Elles sont de types neurologiques, neurocognitives (fréquemment rapportées chez l'adulte), endocriniennes, hématologiques, auditives, ophtalmologiques ou tumeurs radio-induites, d'où la nécessité d'une surveillance multidisciplinaire régulière. Afin d'adapter les modalités thérapeutiques et la qualité de survie, de nombreux facteurs pronostiques ont été rapportés dans la littérature, mais les résultats sont, dans la plupart des temps, contradictoires. Le sexe féminin serait de bon pronostic [7, 15], mais pas pour tous les auteurs [6, 11, 14]. L'hydrocéphalie a été décrite comme un facteur défavorable [16, 17]. Les tumeurs latérales sont associées à un meilleur pronostic. Une infiltration du plancher du quatrième ventricule est un facteur de mauvais pronostique [13]. De même, les tumeurs de stade T1, T2 et T3a sont associées à une meilleure survie par rapport aux celles de stade T3b et T4 [5]. L'influence de la présence de métastases sur le pronostic est controversée [14, 16]. Selon Prados [15], les patients à « risque standard » sont de bon pronostic par rapport aux groupes à « haut risque », mais cette différence n'existe pas dans l’étude prospective de Brandes [5]. Une exérèse chirurgicale complète entraine une meilleure survie [14], mais ceci n'a pas été retrouvé dans toutes les études [13]. Le résidu tumoral postopératoire est aussi une question controversée; il n'a pas d'impact selon Carrie [13], et c'est un facteur de mauvais pronostic selon Chan [14]. Le médulloblastome desmoplasique serait de bon pronostic [7]. L'importance pronostique de la radiothérapie est indiscutable, une dose >50Gy au niveau de la fosse cérébrale postérieure est nécessaire pour une meilleure survie [11, 14]. Enfin, d'autres voies de recherches prometteuses ont démontré, récemment, la présence de marqueurs génétiques associés à un pronostic défavorable (CDK6, la perte de 10q et le gain de 17q) [18]. Dans notre série, devant sa petite taille, aucun de ces facteurs ne s'est révélé statistiquement corrélé à un bon ou mauvais pronostic.

## Conclusion

Le médulloblastome chez l'adulte est moins agressif par rapport à l'enfant. Bien que les progrès dans la recherche fondamentale, des techniques chirurgicales et de radiothérapie ont amélioré sa prise en charge. Une collaboration multidisciplinaire entre neurochirurgiens et oncologues radiothérapeutes est le seul garant d'une amélioration de son pronostic.
